# Catulin reporter marks a heterogeneous population of invasive breast cancer cells with some demonstrating plasticity and participating in vascular mimicry

**DOI:** 10.1038/s41598-022-16802-2

**Published:** 2022-07-25

**Authors:** Mateusz Gielata, Kamila Karpińska, Aleksandra Gwiazdowska, Łukasz Boryń, Agnieszka Kobielak

**Affiliations:** 1 Laboratory of the Molecular Biology of Cancer, Centre of New Technologies, University of Warsaw, S. Banacha 2c, Room 2109, 02-097 Warsaw, Poland; 2 Laboratory of Stem Cells, Tissue Development and Regeneration, Centre of New Technologies, University of Warsaw, Warsaw, Poland

**Keywords:** Breast cancer, Epithelial-mesenchymal transition

## Abstract

Breast cancer is the most commonly diagnosed cancer in women worldwide. The activation of partial or more complete epithelial–mesenchymal transition in cancer cells enhances acquisition of invasive behaviors and expands their generation of cancer stem cells. Increased by EMT plasticity of tumor cells could promote vascular mimicry, a newly defined pattern of tumor microvascularization by which aggressive tumor cells can form vessel-like structures themselves. VM is strongly associated with a poor prognosis, but biological features of tumor cells that form VM remains unknown. Here we show that catulin is expressed in human BC samples and its expression correlates with the tumor progression. Ablation of catulin in hBC cell lines decreases their invasive potential in the 3D assays. Using a novel catulin promoter based reporter we tracked and characterized the small population of invasive BC cells in xenograft model. RNAseq analysis revealed enrichment in genes important for cellular movement, invasion and interestingly for tumor-vasculature interactions. Analysis of tumors unveiled that catulin reporter marks not only invasive cancer cells but also rare population of plastic, MCAM positive cancer cells that participate in vascular mimicry. Ablation of catulin in the xenograft model revealed deregulation of genes involved in cellular movement, and adhesive properties with striking decrease in CD44 which may impact stemness potential, and plasticity of breast cancer cells. These findings show directly that some plastic tumor cells can change the fate into endothelial-like, expressing MCAM and emphasize the importance of catulin in this process and breast cancer progression.

## Introduction

Breast cancer is the most commonly diagnosed cancer in women worldwide^1^, and accounts for 23% of all cancer cases^2^. Breast cancers are categorized into three main groups based on cellular markers reflecting available targeted therapies: Estrogen receptor (ER) or progesterone receptor (PR) positive, Her2 positive and triple negative breast cancer with no ER, PR and Her2 expression having no standard treatment option and the poorest survival^3^. Majority of breast cancer related deaths are a consequence of late diagnosis resulting in resistant to treatment, metastatic disease. The EMT plays a role in many developmental processes, and is also associated with many solid tumors progression. Therefore, pharmacological intervention of this process may represent a crucial therapeutic target. Unfortunately, it is challenging to observe EMT in vivo*,* in human carcinomas. In addition recent studies indicate high heterogeneity within tumor cells undergoing epithelial-mesenchymal transition and exhibiting different phenotypes: epithelial, mesenchymal, or one or more hybrid epithelial-mesenchymal phenotypes within the same tumor^4–7^. This behavior has been reported across different cancer types, and implicated in multiple processes associated with metastasis and appearance of cancer cell subpopulations with most plastic stem cell-like properties. Therefore, labeling, isolation and functional characterization, including cancer stem cell potential, and genetic signature of heterogeneous populations of cancer cells undergoing transient and reversible partial EMT process is very challenging. During breast cancer progression, and metastasis the reduction of intercellular adhesion is one of the critical steps^8^. The process of epithelial to mesenchymal transition is characterized by progressive redistribution or downregulation of apical, and basolateral epithelial-specific tight, and adherens junction proteins, including E-cadherin and α-catenin, and re-expression of mesenchymal molecules like Vimentin and N-cadherin. This switch between relatively stable cell–cell contacts and increase in the motility is necessary for invasion^9^. The important factors playing a role in the EMT process are *TWIST1* and *TWIST2,* responsible for inducing transformation alone or in a cooperation with TGFβ, Wnt, Notch, etc.^10^. Suppression of the E-cadherin transcription is known to be regulated by the Snail1 and Snail2 as well as *Zeb1* and *Zeb2* genes^11^. Downregulation of E-cadherin, and α-catenin is a prognostic marker of poor clinical outcome in majority of solid tumors^12,13^. We observed previously, that loss of cell–cell junction protein α-catenin was accompanied by upregulation of the expression of a new α-catenin homologue, α-catenin-like 1 (α-catulin, catulin)^8^. We also described previously that catulin is highly expressed at the invasion front of human head and neck squamous cell carcinoma (HNSCC). The upregulation of catulin correlated with the transition of tumor cells from an epithelial to mesenchymal state. On the other hand, knockdown of catulin in hHNSCC cell lines dramatically decreased the migratory and invasive potential of those cells in vitro and metastatic potential in vivo*,* in the mouse model. Interestingly, analyses of tumors deficient in catulin showed that its ablation prevented tumor cells from invading the surrounding stroma^14^. Catulin has not been well characterized so far. It was shown to act as a scaffold for the Rho GTPase signaling complex by interacting with the Lbc-Rho GEF^15–17^. Catulin has also been shown to interact with the IKK-b and Lbc to promote tumor cell migration, and resistance to apoptosis^18^. Moreover, it has been shown that catulin knockdown reduces NF-κB, and AP-1 activity, diminishes ERK phosphorylation in malignant melanoma cells, and sensitizes them to treatment with chemotherapeutic drugs^19^. Catulin has also been proven to interact with dystrophin in the dystroglycan-dystrophin/utrophin complex, where dystroglycan mediates cell-ECM adhesion^20–23^. Our laboratory has also described that catulin plays an important role during mouse neural tube closure by acting as a scaffold for RhoA distribution, resulting in proper spatial activation of myosin to influence actin-myosin dynamics, and tension at cell–cell adhesion^24^. Additionally, it has been reported that catulin plays a critical role in cancer metastasis by activating the ILK-mediated Akt-NF-κB-αvβ3 signaling axis^25^. As catulin shows high similarities in structure to vinculin, and α-catenin in N-terminal region that contains binding sites for β-catenin, talin, α-actinin, and actin cytoskeleton^26^ it may act as a cytoskeletal linker protein that modulates adhesive properties, motility and invasive properties of cancer cells.

There is a growing evidence that only a minority of metastasizing cells may persist and form metastases^27–29^. This subpopulation of cells is able to grow, invade, and self-renewal, and exhibit stem-like properties, therefore those cells are called Cancer Stem Cells (CSCs)^30^. CSCs are able to survive even after removal of the primary tumor. This might be due to the mutations and deregulation of epigenetic pathways that favor survival and those may arise from the microenvironment that forces their genetic evolution^31,32^. Tumor microenvironment may provide signals which regulate self-renewal, epithelial-mesenchymal transition, and homeostatic processes such as inflammation, hypoxia and angiogenesis which regulate either entering of CSCs in a dormant state or promoting the reactivation of CSCs that initiate metastasis^33^. Endothelial cells of blood vessels are crucial in cancer progression that is further than delivering oxygen and nutrients^34–36^. Solid tumors that have outgrown beyond a few cubic millimeters to receive nutrients to progress need to induce tumor angiogenesis. Tumor angiogenesis requires development of new blood vessels from established vascular beds and the process is very complex^37–39^. Angiogenesis in tumorigenesis is always pathological, resulting in immature vessels with irregular structure. This hostile environment may facilitate the EMT process and result in the progression and metastases of the primary tumor^40,41^. On the other hand, vasculogenic mimicry is the process where tumor cells mimic endothelial cells and form blood vessel like channels^42–44^. It has been reported among others in breast cancer solid tumors. This process can facilitate cancer cell migration, invasion and metastatic potential as well as resistance to therapies^45,46^. However, the process of tumor cells participation in vascular mimicry is not fully understood yet and needs further investigating.

Here we observe that catulin is expressed in human breast cancer samples, and cell lines, and its expression correlates with the tumor progression. Ablation of catulin in human triple negative breast cancer cell lines decreases their invasive potential in the 3D spheroid, and invasion chip assays. Therefore, to track and characterize the population of invasive breast cancer cells we took advantage of catulin expression and developed a novel catulin promoter based reporter system with green fluorescent protein (GFP) in TNBC cell lines. We verified this system in vitro in 3D spheroid model where catulin-GFP expression correlated with expression of known EMT marker Vimentin. Injection of cells stably expressing catulin GFP reporter plasmid into the immunocompromised mice allowed us to track, isolate and characterized the small population of invasive BC cells. RNAseq analysis revealed enrichment in genes important for cellular movement, cell invasion and interestingly for tumor-vasculature interactions. By analyzing the tumors, we discovered that catulin reporter system marks not only invasive cancer cells enriched at the tumor-stroma border and around newly formed vasculature but also rare population of highly plastic MCAM positive cancer cells that participate in vascular mimicry. We also showed that ablation of catulin in hBC cell lines decreased significantly the invasive potential of those cells in the 3D assays. Analysis of tumor cells in the xenograft model after catulin ablation revealed deregulation of genes involved in cellular movement and adhesive properties. We also observed decrease in membrane localized CD44 protein, which may impact stemness potential and plasticity of breast cancer cells. We confirmed the decrease in CD44 membrane fraction by performing FACS (fluorescence-activated cell sorting) analysis of catulin control and deficient tumor cells. These findings show high intratumoral heterogeneity with population of cells marked by catulin reporter characterized by increased invasiveness as well as plasticity of tumor cells which results in the change of fate into endothelial-like, expressing among other markers, MCAM. Our data confirm the growing number of evidence that cancer cells rarely undergo complete EMT and rather exist in a continuum of E/M intermediate states, preserving high levels of plasticity^47^.

## Results

### Catulin is expressed in human breast cancer samples, and cell lines, and its expression correlates with the tumor progression

**Figure 1:**
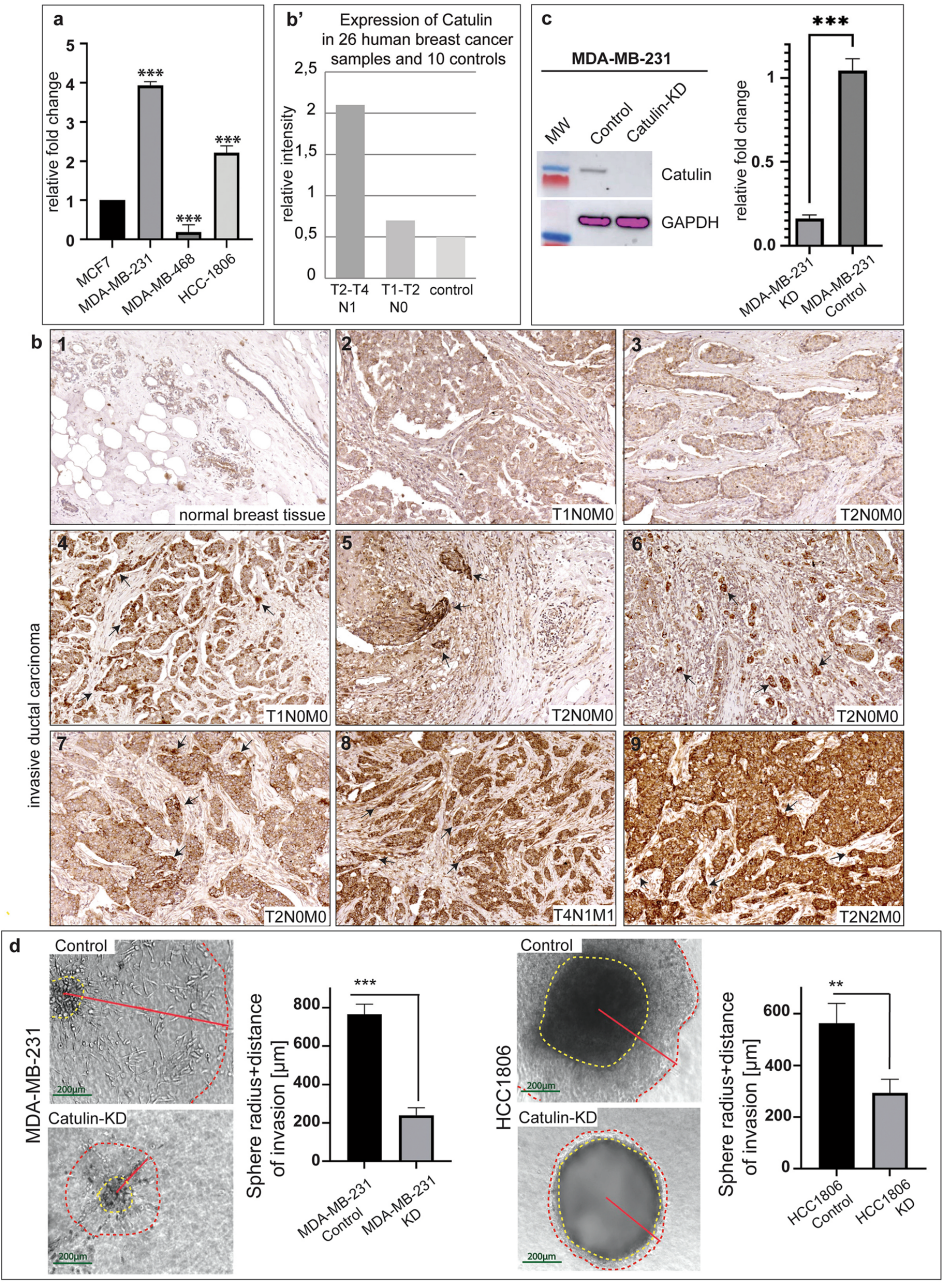
Catulin is expressed in triple negative breast cancer cell lines and in high grade human breast cancer tissue and its ablation in hBC cell lines decreases their invasive potential in the 3D spheroid assay. (**a**), RT-qPCR shows that catulin is upregulated in MDA-MB-231 and HCC1806 cell lines and not in MDA-MB-468 compared to control MCF-7 cell line. (**b**), immunohistochemical analyses show catulin expression in a tissue array slide panel BR8014 (Biomax) consisting of 26 cases of human breast cancer samples (all represented in duplicates) and 10 cancers adjacent or normal breast tissue samples. Tissue origin and tumor grade, as provided by the manufacturer, is indicated. 1–9, representative images are shown. Arrows point out the positive invasive tumor cells. (**b’**), Average expression of catulin in all 26 cases of human breast cancer samples and 10 cancers adjacent or normal breast tissue samples are shown. Tumor samples were divided into two groups: one, advanced group in stages between T2–T4 with lymph nodes involved—N1, second group less advanced in stages between T1–T2 without lymph nodes involved—N0. (**c**), RT-qPCR shows significant stable knockdown of catulin expression in MDA-MB-231 cells (t-test, *p* < 0.005). The results were confirmed on a protein level on Western Blot analysis resulting in no catulin protein in knockdown samples. Presented is cropped gel picture, an original view of the gel is included in Supplementary Fig. S1d). (**d**), 3D spheroid assay shows impaired invasive properties of MDA-MB-231 and HCC1806 knockdown cells compared to control and the calculated distance of invasion is shown on charts and marked with red line. Yellow dotted line represents the sphere body while the red dotted line circles around the invasion zone.

To investigate the level of catulin expression in the triple negative breast cancer cell lines, we used: MDA-MB-231, MDA-MB-468, HCC1806, and non-triple negative MCF-7 cell line as a control. RT-qPCR analysis was performed using pure RNA isolated from those cell lines. As MDA-MB-231 and HCC1806 showed the highest expression level of catulin, we selected those two cell lines for further studies with a main focus on MDA-MB-231 cells (Fig. 1a). To determine catulin protein level in human BC samples, and to check if its expression correlates with tumor progression, we performed immunohistochemical analysis using commercially available tissue array BR8014 from US Biomax (Fig. 1b). It showed different levels of expression in a tissue array consisting of 26 cases of hBC samples (all represented in duplicates), and 10 cancers adjacent, or normal breast tissue samples. There is a minimal catulin expression in the normal epithelium (Fig. 1b1). Low grade tumors, with no lymph nodes involved, showed relatively low expression level of catulin (Fig. 1b2–3). Interestingly, in some samples, stages T1–T2, catulin expression was observed in tumor cells or small clusters of cells, invading tumor stroma (arrows in Fig. 1b4–7). High level of catulin expression was noticed in stages T2–T4, especially in samples with lymph nodes involved (arrows in Fig. 1b8–9). In Fig. 1b’, average expression of catulin in all 26 cases of hBC samples, and 10 cancers adjacent or normal breast tissue samples are shown. Tumor samples were divided into two groups: first, advanced group in stages between T2–T4 with lymph nodes involved—N1, second group less advanced in stages between T1–T2 without lymph nodes involved—N0. The highest expression of catulin was noticed in stages T2–T4 with lymph nodes involved, 4 times higher compared to stages T1–T4 without lymph nodes involved (Fig. 1b’ and Sup. Fig. 1b). Tumor grade, as provided by the manufacturer, is indicated in Sup. Fig. 1b. These suggest that catulin protein level is important for highly aggressive breast cancer cells and its expression correlates with the tumor progression.

**Figure 2:**
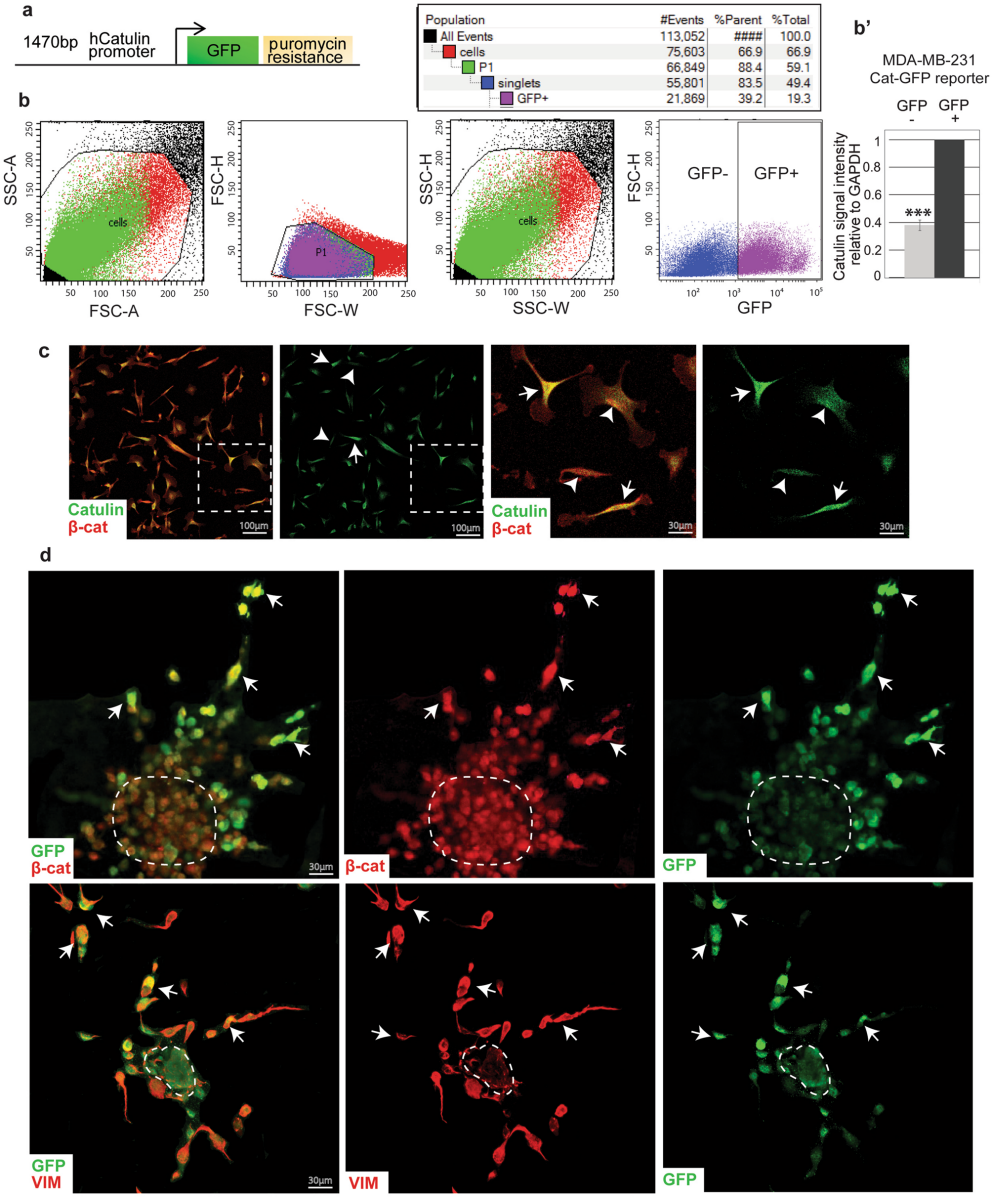
Development of catulin promoter based novel reporter system to label and track population of invasive cancer cells of breast cancer. (**a**), Shown is the graphical representation of catulin promoter novel reporter system where GFP expression is driven from catulin promoter expression. (**b**), Flow cytometry analysis of MDA-MB-231 cells transfected with catulin promoter reporter system. Gating strategy set to determine population of GFP positive cells. (**b’**), RT-qPCR shows difference in catulin expression level in sorted MDA-MB-231-Cat-GFP + and MDA-MB-231-Cat-GFP- cells (t-test, *p* < 0,005). (**c**), Immunostaining of MDA-MB-231cells using antibodies against catulin and beta-catenin, dashed squares in left panels indicate magnified panels on the right side. Arrows indicate cells with strong catulin expression, whereas arrowheads indicate cells with lower catulin expression. (**d**), Spheres formed from MDA-MB-231-Cat-GFP reporter cell line were analyzed on immunofluorescence. In all sets there was GFP stained in green and in red stained was β-catenin and Vimentin. On the right side one can find the magnification of spheres with green and red signal colocalization. Arrows indicate invasive cells spreading into the matrigel, whereas dashed line indicates sphere body.

**Figure 3:**
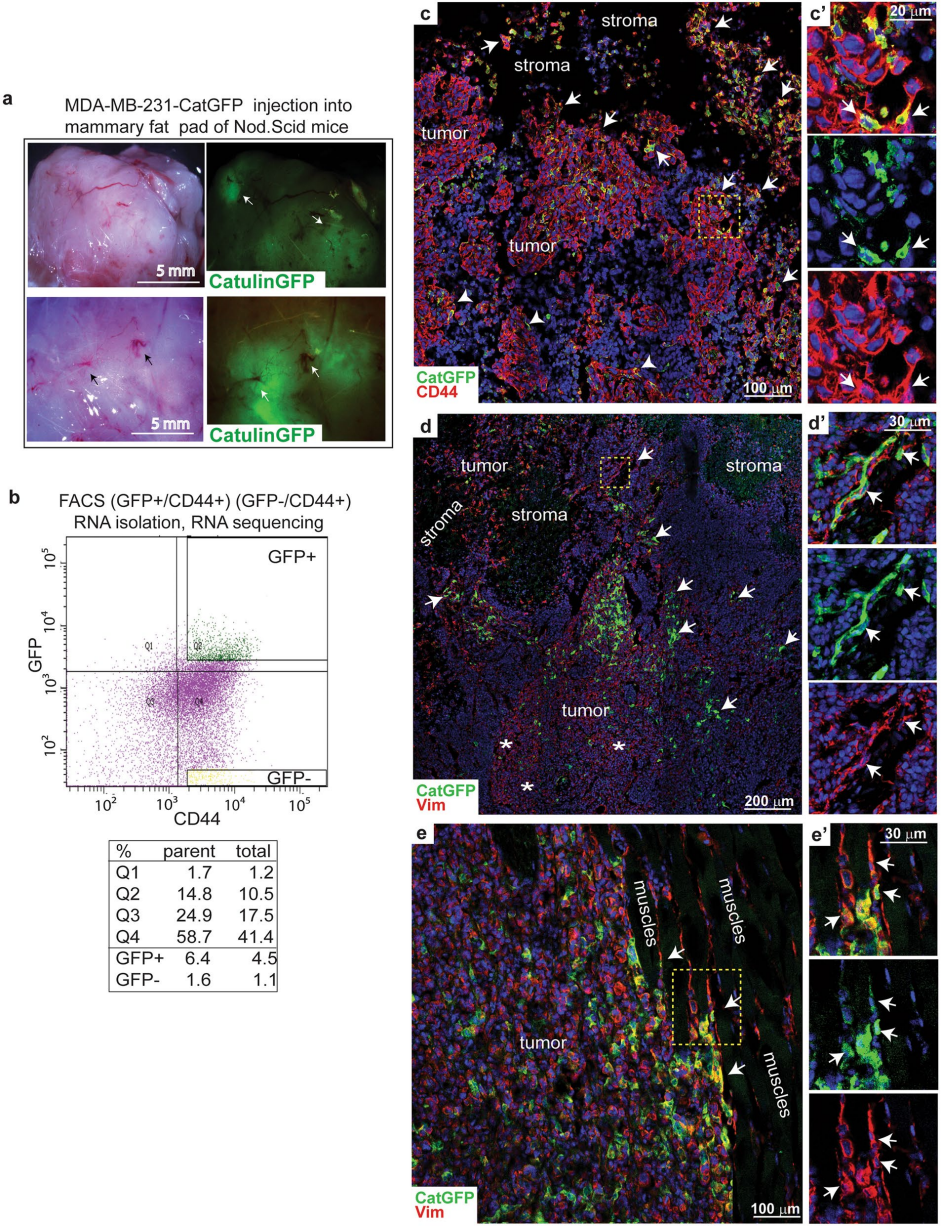
Catulin-GFP reporter system marks the population of tumor cells at the invasive front in the xenograft model of breast cancer enriched with the vasculature. (**a**), MDA-MB-231-Cat-GFP reporter cell line was injected into the mammary fat pad of Nod.Scid mice. Tumors were imaged for GFP. Arrows show the vasculature network enrichment. (**b**), Tumors formed after injection of MDA-MB-231-Cat-GFP cells were sorted for GFP +/CD44 + cells and GFP −/CD44 +. RNA was isolated and used for RNAseq analysis. (**c**), Isolated tumors after injection of MDA-MB-231-Cat-GFP reporter cell line into the mammary fat pad of Nod.Scid mice were cut and stained for GFP (green), and epithelial marker –CD44 (red). Nuclei were stained with DAPI (blue). Arrows indicate cells highly expressing catulin (GFP +) at the tumor-stroma invasive front. Arrowheads point out cells expressing catulin (GFP +) in the patches of tumor cells located deeper in the tumor but still mixed with the stroma. In the yellow bracket marked is the area enlarged on (**c’**)—where cells highly expressing GFP are shown and marked with arrows to be simultaneously expressing CD44 (red). (**d**), Isolated tumors after injection of MDA-MB-231-Cat-GFP reporter cell line into the mammary fat pad of Nod.Scid mice were cut and stained for GFP (green), and mesenchymal marker -Vimentin (red). Nuclei were stained with DAPI (blue). Arrows indicate cells highly expressing catulin (GFP +) at the tumor-stroma invasive front. Asterisks point out broad vimentin expression. In the yellow bracket marked is the area enlarged on (**d’**)—where cells highly expressing GFP are shown and marked with arrows to be simultaneously highly expressing signal from vimentin (red). (**e**), Demonstrated is the border of the tumor and muscles, enriched in GFP + cells. In the yellow bracket marked is the area enlarged on (**e’**)—where GFP + cells squeezed in between muscle cells also co-express red signal from vimentin (marked with arrows).

**Figure 4:**
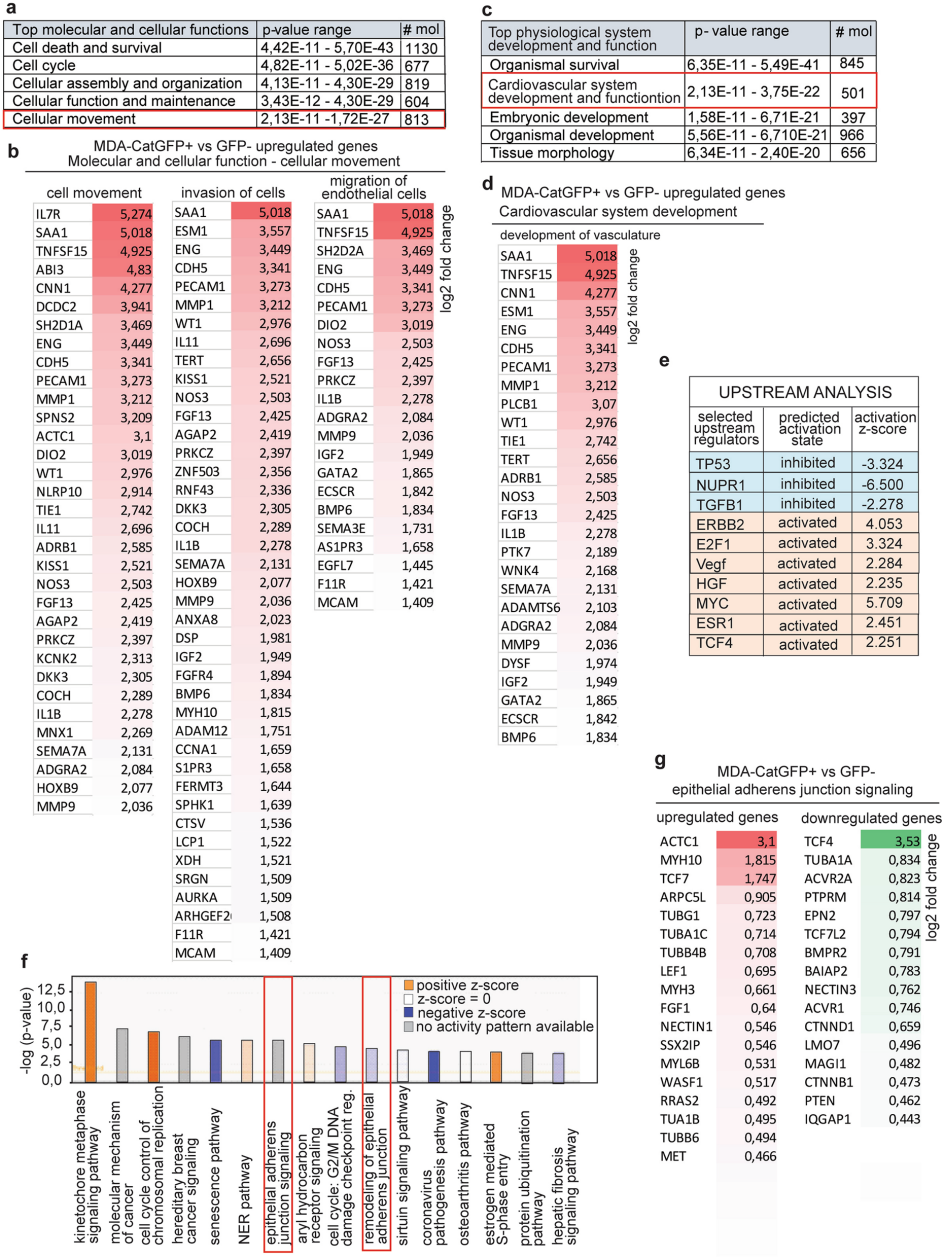
RNAseq and Ingenuity Pathway Analysis of invasive cancer cells (GFP +) reveals enrichment in genes responsible for cell movement and vasculogenesis in cancer. (**a**), Top 5 deregulated categories of molecular and cellular functions. In the red bracket enlightened is the most relevant cellular movement function. (**b**), listed are genes upregulated (log2 fold change > 1.4) in categories of cell movement, invasion of cells and migration of endothelial cells. (**c**), Top 5 deregulated categories of physiological system development and function. In the red bracket, enlightened is the cardiovascular system development and function. (**d**), Upregulated genes involved in development of vasculature. (**e**), Upstream analysis revealed top molecular upstream regulators with predicted activation state and z-score. (**f**), Top deregulated canonical pathways. In red brackets marked are pathways involved in adherens junction signaling and remodeling important for tumor progression. Orange indicates activation of the pathway; blue indicates inhibition and gray indicates no activity pattern available for this particular pathway in IPA. Gradient in colors is reflecting the level of activation or inhibition. (**g**), Listed are genes upregulated and downregulated in epithelial adherens junction signaling pathway with log2 fold change > 0.4.

### Ablation of catulin in hBC cell lines decreases their invasive potential in the 3D spheroid assay

Seeing that catulin is particularly upregulated in highly invasive triple negative breast cancer cell lines, and its expression correlated with the tumor progression, we wanted to investigate if catulin knockdown would influence invasive potential of those cells in vitro. To obtain stable TNBC cell lines deficient in catulin, we generated lentivirus containing catulin-specific shRNA to knockdown catulin in BC cell lines MDA-MB-231 and HCC1806. We were able to successfully knockdown (KD) catulin in the MDA-MB-231 (G85) and HCC1806 (G85), compared to cells transduced with the non-silencing control (GNS) on the RNA and protein level (Fig. 1c and Sup. Fig. 1a). To evaluate the invasive potential in vitro we used 3D spheroid assays. Formed spheres were allowed to invade matrigel-collagen stroma, and distance of invasion was calculated. We noticed, that for both MDA-MB-231, and HCC1806 cell lines, morphology of G85 KD spheres remained unchanged, however the distance of invasion was impaired in G85 KD spheres compared to GNS controls (Fig. 1d). Mean sphere distance of invasion for MDA-MB-231 GNS control cells was close to 800 µm compared to 200 µm for G85 KD cells, and for HCC1806 GNS control cells 550 µm compared to 300 µm for G85 KD cells (Fig. 1d). Those results correlate with previous findings where SCC cells with downregulated catulin level were also unable to efficiently migrate and invade in vitro in 2D migration and invasion assays^14^. To confirm the results in the independent assay, we took the advantage of 3D cell culture invasion chips. 10^6^ of MDA-MB-231 G85 KD and GNS control cells were seeded in the media channel of a chip. At the time point 0 h we observed that GNS cells group together, and gather close to the medium-matrigel stroma barrier compared to G85 KD cells that were dispersed in medium (Fig. Supl. 1c). After 96 h we calculated the amount of cells that passed the barrier, and invaded matrigel stroma. As shown in the Fig.Supl. 1c total mean of the number of invasive cells was estimated around 140 for GNS control, and around 50 for G85 KD cells. In addition, classical in vitro migration, and invasion assays using this cell line, again showed that catulin ablation decreased the ability of these cells to migrate, and invade (Supplementary Fig. S2) in^14^. All these data suggest that catulin is an important factor determining invasive potential in human triple negative breast cancer cell lines in vitro and lack of this protein halts their invasive potential.

### Labeling and tracking of invasive breast cancer cells using novel catulin promoter based reporter system

We described previously that catulin marks the invasive front of SCC, and that upregulation of catulin expression correlates with the transition of cells from epithelial to mesenchymal morphology^8,14^. Moreover, we have already shown that catulin expression correlates with BC progression and cancer cells invasive potential. Taken together, these data suggest that catulin might be a great marker of invasive, and motile cancer cells. To mark those cells, we developed a novel catulin promoter based reporter system, in which GFP expression is driven directly from catulin short promoter (Fig. 2a). After stable transfection of MDA-MB-231 cell line with catulin reporter plasmid, followed by puromycin selection, GFP fluorescence of established MDA-MB-231 CatGFP cell line was analyzed using flow cytometry. We gated for GFP + signal comparing reporter cell line to corresponding original MDA-MB-231 cell line as a negative control (representative results are shown in Fig. 2b). Around 20% of total cells (40% of parent) highly expressed GFP signal, coming from catulin promoter, and this percentage was stable in culture. To test the correlation of GFP fluorescence with catulin expression level, we performed qPCR analysis of catulin expression in sorted MDA-MB-231GFP + and MDA-MB-231GFP- populations (Fig. 2b’). This analysis confirmed enrichment of catulin expression in MDA-MB-231GFP + population. To verify how heterogeneous, the catulin expression is in the MDA-MB-231 cell line, we performed immunostaining of cells using antibodies against catulin and beta-catenin (general epithelial marker). As expected, we observed differences in the level of catulin expression (Fig. 2c). To check in vitro the localization of cells with GFP expression in the reporter system, we used 3D sphere invasion model, and immunostained the spheres with GFP antibody, and known EMT marker, Vimentin as well as cell–cell adhesion, and Wnt signaling pathway marker, β-catenin (Fig. 2d). The most outer cells, located at the invasion front of the sphere show the strongest GFP signal, and this signal correlates with the expression of EMT marker, Vimentin (arrows in Fig. 2d, lower panel). In both cases GFP and Vimentin signal is much weaker in the sphere body, in the center (dashed line). The strongest expression of GFP signal is also visible in the cells invading into the matrigel from the reporter sphere stained with β-catenin (arrows in Fig. 2d, top panel). In this case GFP signal is also much weaker in the sphere body, whereas β-catenin expression is quite uniform (dashed line). These data support the idea that novel Catulin-GFP reporter system marks invasive and motile cells in vitro*,* in the 3D model.

### Genetic signature of Catulin GFP positive breast cancer cells by RNAseq analysis revealed enrichment in genes important for migration, invasion and tumor-vasculature interactions

As we developed a functioning reporter system marking invasive BC cells, we wanted to check the genetic signature of those cells. We used in vivo xenograft model, where the MDA-MB-231-Cat-GFP reporter cell line was injected into the mammary fat pad of 5 Nod.Scid mice. Primary tumors were formed after 8 weeks and exhibited patchy signal for the GFP. Interestingly, visible vasculature was located in the GFP positive areas of the tumor mass (arrows in Fig. 3a). Tumors after dissection were collected, either for further immunohistochemical analysis, or sorted in order to collect GFP + and GFP −  fraction for RNAseq analysis. To correlate localization of invasive cells (GFP +) with known EMT marker Vimentin, and epithelial marker CD44, we performed immunohistochemical staining of tumors formed after the injection of the MDA-MB-231-Cat-GFP reporter cell line. Strong GFP fluorescence, indicating catulin GFP reporter expression, was observed in the areas of tumor-stroma interface, especially in the cells invading surrounding stroma (arrows in Fig. 3c,d and e). On the other hand, Vimentin was expressed relatively broadly in the tumor (stars in Fig. 3d), not only at the tumor invasion front (arrows in Fig. 3d,e). Catulin GFP + cells marked smaller population of cancer cells with the majority of them co-expressing Vimentin (arrows in Fig. 3d, d’ and e, e’), and the level of Vimentin expression in GFP positive cells, at the tumor invasion front, was much higher than in the tumor center. The strong correlation of the Vimentin and GFP expression is presented in magnified areas of tumor cells invading into the stroma (arrows in Fig. 3d’), and into the muscles (arrows in Fig. 3e’). This shows high heterogeneity within tumor mass with Catulin-GFP reporter, marking tumor cells with the highest Vimentin expression. Co-staining of CatulinGFP reporter derived tumor with the human specific CD44 antibody also shows similar pattern of GFP expression, with majority of GFP + cells localized at the tumor stroma interface (Fig. 3c and c’). To determine the genetic signature of the catulin GFP + cells we isolated, and FACS sorted GFP + CD44 + and GFP-CD44 + cells from 3 independent tumors formed after the injection of the MDA-MB-231-Cat-GFP reporter cell line (Fig. 3b) using CD44 as an additional tumor cell marker to increase sort purity. RNAseq analysis was performed comparing RNA isolated from GFP + /CD44 + versus GFP−/CD44 + sorted cells, and proper function of the reporter system and sorting strategy was confirmed by the appearance in the RNAseq data CTNNAL1 gene in CatGFP + /CD44 + population as an internal control (log2FC 0.43, *p*val 0.04). Comparison of RNA from GFP + /CD44 + versus GFP-/CD44 + cells revealed a list of 1856 upregulated, and 1617 downregulated genes (*p* < 0.05). The minimum log2-fold change considered to be significant change in the expression between the two populations was set up at log2FC 0.43, *p*val ≥ 0.05). Principal component analysis (PCA) and Volcano plot of RNAseq analysis for the MDACatGFP + /CD44 + (GFPp), and the MDACatGFP −/D44 + (GFPn) sorted populations is presented in Supplementary Fig. 3a. The Ingenuity Pathway Analysis (Qiagen), revealed cellular movement to be deregulated as one of the top 5 molecular and cellular functions. It includes 813 deregulated molecules (Fig. 4a). Interestingly, genes upregulated in this category were included in subcategories revolving around cell movement, invasion of cells, and migration of endothelial cells, where particularly interesting were genes like *SAA1, ENG, CDH5, PECAM1, DIO2, NOS3, MMP9* being responsible for tumor vascularization process (Fig. 4b). Then we analyzed top physiological system development and functions. Top 5 categories included cardiovascular system development and function, having 501 molecules deregulated (Fig. 4c). Subcategory of development of vasculature revealed same upregulated genes that were shown in Fig. 4b, *SAA1, ENG, CDH5, PECAM1, DIO2, NOS3, MMP9,* and additionally *WT1, TIE1, GATA2,* and others (Fig. 4d). Upstream analysis showed *Vegf, HGF* and *MYC* among others as a potential upstream regulators of observed gene changes (Fig. 4e). Interestingly, top pathways deregulated in the catulin GFP + cells involved epithelial adherens junctions signaling and remodeling of epithelial adherens junctions (Fig. 4f) with upregulation of Wnt pathway transcription factors* Tcf7, Lef1* as well as *Fgf1 and Met* (Fig. 4g). These data suggest that increased expression of catulin in the invasive cancer cells correlates with the expression of genes involved in migration, and also could be important in the modulation of adhesive properties of cancer cells and their interactions with vasculature.

### Invasive breast cancer cells express endothelial marker MCAM, and participate in vascular mimicry

**Figure 5:**
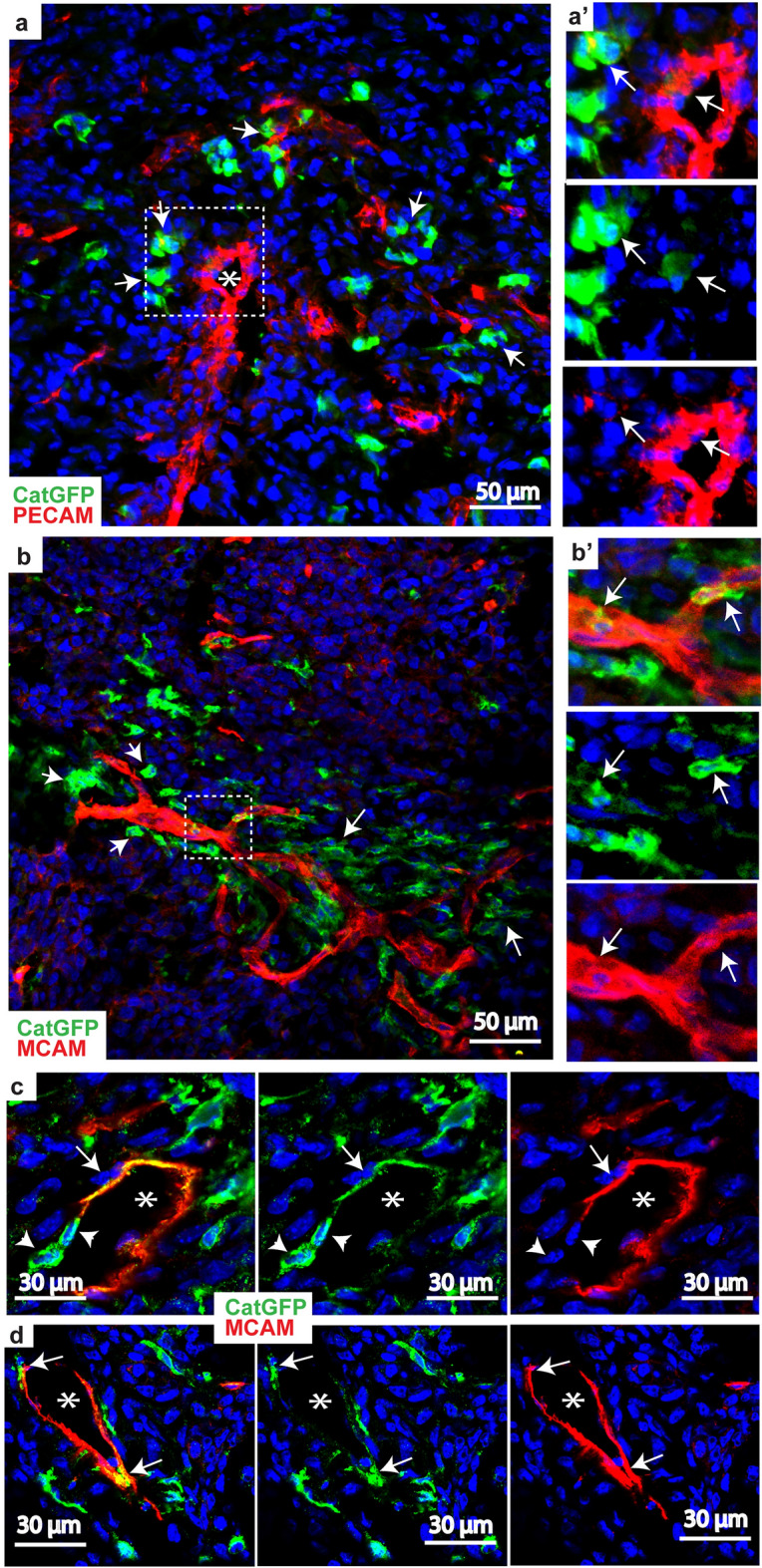
Catulin positive breast cancer cells localize in the areas enriched in vasculature and coexpress endothelial marker—MCAM (CD146). (**a**), Immunostaining of tumor isolated after injection of MDA231-Cat-GFP cells. In green shown are highly invasive cancer cells localized in the areas enriched in vasculature stained with endothelial specific marker PECAM in red. Nuclei are stained with DAPI (blue). With arrows marked are GFP positive cells and with asterisk blood vessel. (**a’**), Enlarged, is the area presented in white bracket. With arrows marked are cells both expressing GFP and PECAM. (**b**), Immunostaining of tumor isolated after injection of MDA231-Cat-GFP cells. In green shown are cancer cells localized in the areas enriched in vasculature stained with endothelial specific marker MCAM in red. Nuclei are stained with DAPI (blue). With arrows marked are GFP positive cells. (**b’**), Enlarged, is the area presented in white bracket. With arrows marked are cells both expressing GFP and MCAM. (**c**,**d**), Two examples of vascular mimicry. With arrows marked are cells expressing both GFP + (green) and endothelial marker MCAM (red). Arrowheads indicate GFP + cells which are MCAM negative but still participate in vascular mimicry. With stars marked is the lumen of the vasculature.

As our RNAseq analysis of the catulin reporter GFP + breast cancer cells revealed enrichment in genes important for tumor-vasculature interactions, we stained isolated tumor samples with antibody against PECAM (CD31)—well known endothelial marker. We noticed that indeed some of the Catulin GFP + cells localize in the areas rich in endothelial structures, stained in red (stars in Fig. 5a) attaching themselves to the endothelial walls at first glance. Interestingly, some of the GFP + cancer cells co-expressed GFP and endothelial marker PECAM (arrows in Fig. 5a and a’). Next we focused on the MCAM (CD146) which was also upregulated in RNAseq data, because CD146 is not only an adhesion molecule on endothelial cells, but also a cellular surface receptor of different ligands, actively involved in signaling in the numerous physiological and pathological processes. Overexpression of CD146 can be observed in most of malignancies, and is implicated in the progression of cancers, especially vascular, and lymphatic metastasis. Interestingly, we observed that marked by GFP invasive breast cancer cells were enriched at the proximity of the CD146 expressing vascular structures (arrows in Fig. 5b). Sometimes, we observed that those GFP + cancer cells expressed both GFP and CD146 and even participated in the formation of single layered vascular like structures (arrows in Fig. 5b’, c and d). We also noticed GFP +/MCAM − cancer cells that participated in vascular mimicry together with GFP +/MCAM + cells, as marked by arrowheads in Fig. 5c. This suggests, that invasive cancer cells can participate in vascular mimicry process, and also with increased plasticity start to exhibit endothelial fate and express endothelium specific markers like MCAM, resembling vascular structures.

### Signaling pathways affected by the decrease of catulin expression in BC

**Figure 6:**
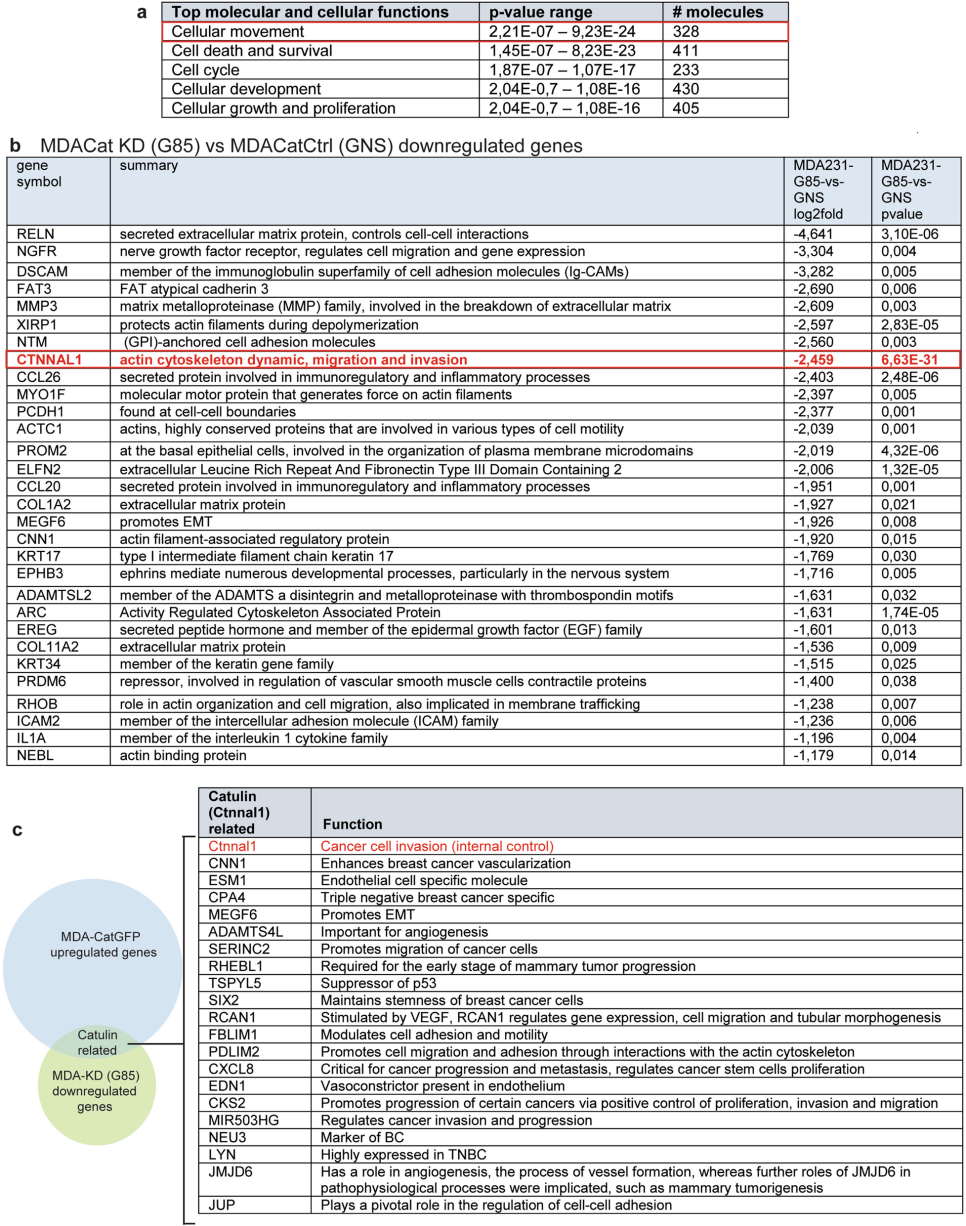
RNAseq and Ingenuity Pathway analysis of catulin KD tumor cells reveals deregulated genes involved in cellular movement. (**a**), Top 5 molecular and cellular functions show cellular movement to be highly deregulated in case of catulin KD. (**b**), List of genes highly downregulated in catulin KD cells derived from tumors. In the red bracket highlighted is catulin as an internal control. (**c**), Biovenn analysis of a common set of genes to be both highly upregulated in catulin reporter GFP + cells and highly downregulated in catulin KD tumor cells. Set of catulin related genes listed on the right side of the panel.

To better understand how the ablation of catulin in tumors influence the capability of those cells to invade, change adhesive properties, and plasticity we performed RNAseq analysis of tumor cells isolated using FACS, after injection of NOD.Scid mice with the MDA-MB-231 G85 (catulin KD) and the GNS (Ctrl) cell lines (Fig. 6). Principal component analysis (PCA) and Volcano plot of RNAseq analysis for the MDA-MB-231 G85 (catulin KD) and the GNS (ctrl) sorted populations is presented in the Supplementary Fig. 3b. We performed functional annotation of the microarray data using Ingenuity Pathways Software to identify the biological functions that were significantly represented in the catulin-deficient tumors. When we compared catulin KD versus control group, we got a list of 730 downregulated (internal control *CTNNAL1* gene downregulated (log2fold change − 2459) and 528 upregulated genes (Fig. Supl. 3b). Ingenuity Pathway analysis revealed that cellular movement was on the top of 5 deregulated molecular, and cellular functions (Fig. 6a). The list of 30 most downregulated genes in catulin knock down cells included genes involved in cellular movement (*NGFR, XIRP1, MYO1F, ACTC1, *etc.), cellular adhesion (*DSCAM, NTM, PCDG1, ICAM2 *etc*.*), cell-ECM interaction (*MMP3, COL1A2, PDLIM2, *etc*.*), EMT (*MEGF6*) and migration of cancer cells (*SERINC2, MIR503HG, HSPB1, *etc*.*) (Fig. 6b). Those results emphasize the role of catulin in cellular movement, EMT, interactions with microenvironment, and progression of cancer, as downregulation of this gene resulted in deregulation of genes important for those processes. As we were mostly interested in the upregulated genes in the invasive catulin reporter GFP + cells, we wanted to evaluate if any of these genes appear in the downregulated gene set from catulin KD cells. We used Biovenn web tool to compare and visualize those sets of genes^48^. 75 genes (catulin related) appeared to be both highly upregulated in the reporter Catulin GFP + invasive tumor cells and highly downregulated in catulin KD tumor cells. From those 75 genes we selected 21 to be involved in vascularization, TNBC development and EMT related. As shown in the Fig. 6c we found internal control *CTNNAL1* gene to be present in both groups. Interestingly, in these 75 genes many were responsible for tumor vascularization process—*CNN1, ESM1, ADAMTSL4, RCAN1, EDN1, JMJD6, JUP.* Some were specific for TNBC development—*CPA4, SIX2, NEU3, LYN,* and some were specific for inducing cancer migration, EMT and metastasis—*MEGF6, SERINC2, RHEBL1, FBLIM1, CXCL8, CKS2.* This suggests that catulin might play a role in the vascularization process, TNBC development, cancer cell migration and metastasis.

### Catulin influences breast cancer cells adhesive properties and stem cell potential

**Figure 7:**
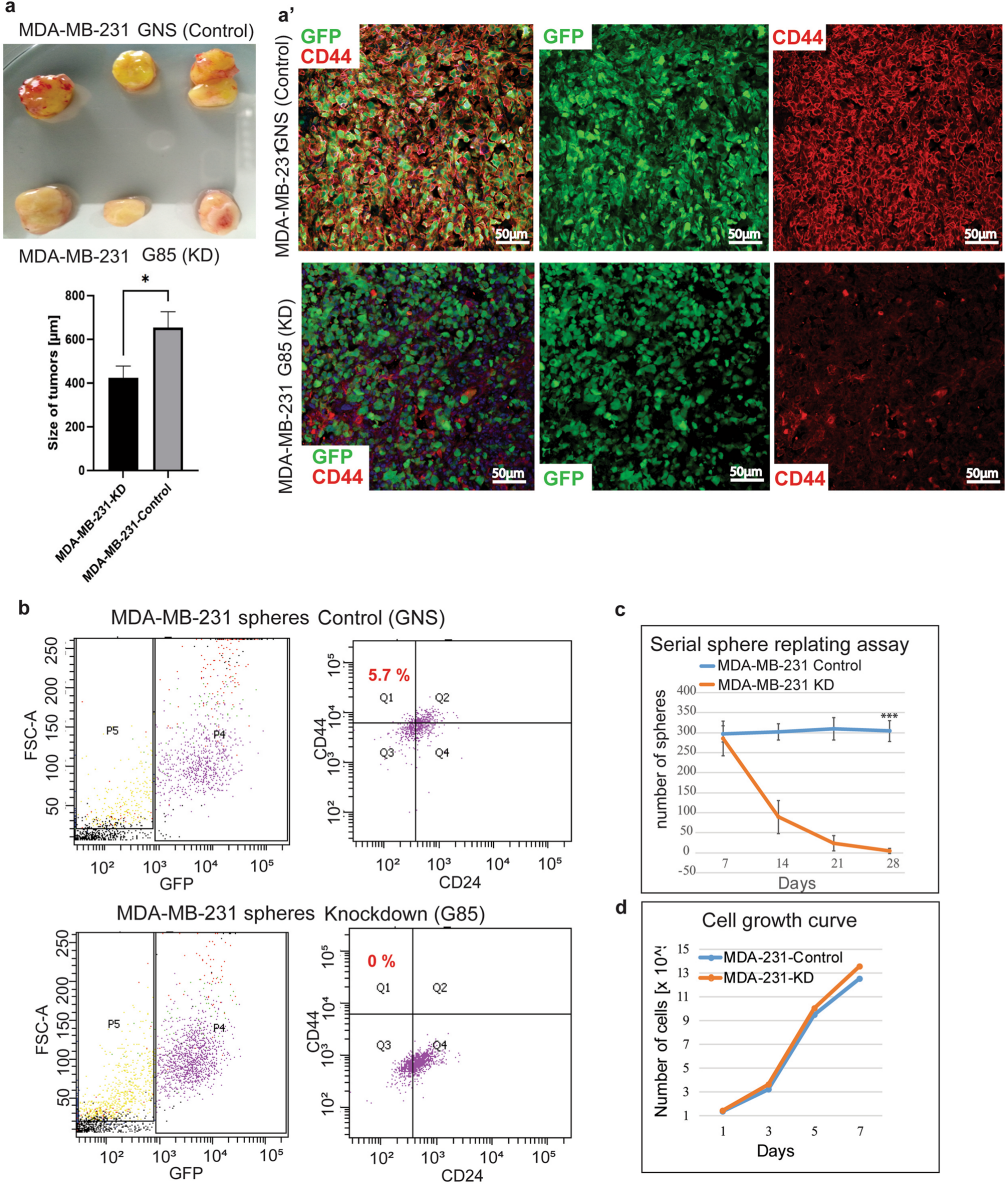
Catulin influences breast cancer stem cell potential. (**a**), Tumors formed after injection of MDA-MB-231-Cat-GNS (control) and MDA-MB-231-G85 (catulin KD) cells. Tumors size was measured and data are presented as a graph. (**a’**), Tumors formed after injection of MDA-MB-231-Cat-GNS (control) and MDA-MB-231-G85 (catulin KD) cells were cut and stained for GFP (green) and CD44 (red). (**b**), Flow cytometry analysis of breast cancer stem cells markers CD44 + /CD24 − of MDA-MB-231 GNS (control) and G85 (catulin KD) sphere cells (the sort was repeated twice). (**c**), Sphere serial replating assay of MDA-MB-231-Cat-GNS (control) and MDA-MB-231-G85 (catulin KD) cells (done in triplicate). Sphere forming capacity presented as a number of spheres generated on each time point. (**d**), Growth curve of MDA-MB-231-Cat-GNS (control) and MDA-MB-231-G85 (catulin KD) cells (done in triplicate).

High expression of CD44 and low expression of CD24 are well-known breast cancer stem cells markers^49^. To further investigate the possible increase in the plasticity of catulin expressing cells we wanted to verify if CD44 expression correlates with the expression of catulin in vivo. Therefore, we analyzed tumors formed after injection of NOD.Scid mice with the MDA-MB-231 G85 (catulin KD) and the GNS (Ctrl) cell lines. We observed small difference in the tumor size between WT and Catulin KD (Fig. 7a). The slides with sections cut from those tumors, were immunostained using human specific CD44 antibody. The level of CD44 expression is different in tumor cells, however most of the control tumor cells are positive for CD44 expression (Fig. 7a’ upper panel). However, when we compared catulin depleted tumors (G85) with control (GNS) we noticed decrease in the CD44 expression (Fig. 7a’, lower panel). As breast cancer stem cells are characterized as CD44 + /CD24 −  we trypsinized the MDA-MB-231 G85 and GNS spheres, stained them with CD44 and CD24 specific antibodies, and analyzed by flow cytometry. As it can be seen in the Fig. 7b, there is a tremendous downshift in CD44 localized at the membrane in the catulin ablated cells, while the CD24 expression remains similar in both groups. These data suggest that knockdown of catulin correlates with the decrease in the CD44, and may impact stemness potential of breast cancer cells. To verify that this deregulation of CD44 expression is linked to impaired stemness potential, we performed sphere serial re-plating assay. In this method spheres, every 7 days are subjected to serial passage allowing to determine the number of cancer stem cells with each passage^50^. As it can be seen in the Fig. 7c, sphere forming capacity for G85 (catulin KD) number of spheres dropped dramatically over the time, compared to GNS control. Similar results were obtained for the HCC1806 cell line (Fig. Supl 2). On the other hand, growth curve performed using MDA-MB-231 G85 and GNS cells in the 2D culture conditions didn’t show any difference in proliferation (Fig. 7d) indicating that the function of catulin might be important for proper adhesion and/or apical polarity regulation as described previously^24^. Together these results suggest that catulin might be involved in modulation of cellular adhesive properties which is important for stem cell function.

## Discussion

Tumor progression, invasion and metastatic dissemination is a very complex process requiring vast variety of transcriptional and protein function changes. Epithelial-to-mesenchymal transition allows to destabilize cadherin/catenin cell–cell contacts and transform epithelial state of a cell into mesenchymal one. It would not be possible without cell-stroma interactions. Catulin, α-catenin homologue has been previously described to be upregulated at the invasion front of human squamous cell carcinoma resulting in an increased motility and progression of those tumors^14^. Here we observe that catulin is highly expressed in the triple negative breast cancer cell lines. Moreover, elevated catulin expression was noticed in human tissue array and its expanding level correlated with the tumor progression. Additionally, knockdown of catulin in TNBC cell lines resulted in a decreased migratory and invasive potential on 3D spheroid model and in invasion chip assay what correlates with the previously described results performed on HNSCC cells in our laboratory^14^. It has also been previously shown that catulin promotes cell migration and invasion of lung carcinoma cells. Catulin interacts directly with ILK, what activates ILK/Akt/NF-κB signaling pathway. This led to the upregulation of fibronectin and integrin α_v_β_3_ what resulted in cancer metastasis^25^. Same signaling cascade occurs probably in breast carcinoma progression. Hence inhibiting of catulin expression leads to decreased motility and invasiveness of TNBC cells. Basing on the fact that the higher invasive potential of cancer cells the higher catulin expression and the fact that depleting catulin decreases aggressiveness of those cells we propagate that catulin is a great marker for invasive breast cancer cells. By developing novel reporter system based on catulin promoter-GFP we were able to characterize and track highly invasive cells. We demonstrated that this population of breast cancer cells exhibit mesenchymal as well as cancer stem cell features, like high CD44 expression in vitro in spheroid model. Very interesting was the finding that knockdown of catulin results in the significant attenuation of CD44 signal on the membrane, when analyzing cells on flow cytometry and in tumor tissue from xenograft transplant. These results suggest that this fraction of invasive cells highly expressing catulin and CD44 + may constitute so called Cancer Stem Cells—a subpopulation of tumor cells having tumor-initiating properties as well as being able to reconstitute the cellular heterogeneity typical of their tumors of origin^51^, which we are planning to test further. Potential role of catulin in stem cells, in this case in the adult stem cells, was shown in the work of S.J. Morrison group. They showed the localization of haematopoietic stem cells (HSCs) which reside in a perivascular niche using a green fluorescent protein (GFP) knock-in for the catulin gene in mice, discovering that catulin(GFP) is expressed by only 0.02% of bone marrow haematopoietic cells, including almost all HSCs^52^. It will be interesting to further test the role of catulin in adhesive properties of stem cells and cancer stem cells. Our RNAseq data also indicated elevated level of MMP-9 expression in Catulin GFP + cells. It has been observed that CD44 co-localizes with active MMP-9, what promotes degradation of extracellular matrix and facilitates invasion^53^. This might be one of the mechanisms driving high invasive potential of those cells however interaction between catulin and CD44 pathway needs further investigating, in terms of cancer propagation. Transcriptional profiling of invasive triple negative breast cancer cells, basing on our novel reporter system, revealed significant upregulation of genes involved in cell motility, invasive potential and angiogenesis. Many of the genes we listed above, have been already associated with tumor vascularization propagation in other types of cancer—*PECAM1, ESM1, CD146, CDH5, ENG, ICAM4, PTN, FGF1, ANKRD1, GATA2, KDR, NOS3, SERPINE1, TIE1*^54,55^. Interestingly when we compared genes upregulated in the invasive TNBC cells with downregulated genes from catulin KD samples, we got a list of 75 genes including genes specific for vasculogenesis *CNN1, ESM1, ADAMTSL4, RCAN1, EDN1, JMJD6, JUP.* Some were specific for TNBC development—*CPA4, SIX2, NEU3, LYN,* and some were specific for inducing cancer migration, EMT and metastases—*MEGF6, SERINC2, RHEBL1, FBLIM1, CXCL8, CKS2.*

We recently used the same reporter system in the head and neck squamous cell carcinoma model, which is characterized by different way of invasion and spreading than breast cancer. HNSCC spreads mostly by loco-regional invasion, moving in clusters, very often along innervation^56^. Molecular profiling of CatulinGFP + reporter cells from the HNSCC model also provides a list of genes associated with cells movement and invasion. Interestingly transcriptional profile of CatulinGFP + reporter cells from HNSCC model overlapped with the expression of signature genes from single cell analysis of human HNSCC specimens, related to partial EMT, so hybrid between epithelial and mesenchymal state rather than complete EMT. In addition, we also observed upregulation of genes with adhesive properties, involved in the axonal guidance like L1CAM, Neuropilin-1, semaphorins, and ephrins, emphasizing potential interactions of cancer cells and neuronal components of the stroma^56^.

Our data from both systems implicate that catulin may play a pivotal role in cancer type specific tumor-microenvironment interaction, being involved in modulation of adhesive properties of tumor cells. The possible mechanism of increased catulin expression in invasive cancer cells comes from recent paper by Cassandri et al.^57^. They showed that zinc-finger protein 750 (ZNF750) is a negative regulator of the migration, and invasion of breast cancer cells by repressing a prometastatic transcriptional program, which includes genes involved in focal adhesion and extracellular matrix interactions, such as LAMB3 and CTNNAL1. The expression of CTNNAL1 and LAMB3 inversely correlated with ZNF750 expression in breast cancer. ZNF750 is responsible for recruitment of the epigenetic modifiers KDM1A and HDAC1 to the promoter regions of LAMB3 and CTNNAL1, affecting histone marks and trans activating these genomic sites. Importantly, they also showed gene expression analysis in cancer patient datasets which indicated that ZNF750, and its targets were negative prognostic factors in breast cancer.

The change in adhesive properties, resulting in increased plasticity of those cells might be also important for the switch of tumor cells into endothelial phenotype and participation in the vascular mimicry. Two different concepts at first glance which are Cancer Stem Cells and Vascular Mimicry start to merge together having influence on each other. It has been described that Cancer Stem Cells stimulate Vascular Mimicry in the microenvironment of the tumor, by differentiating of endothelium cells together with cancer cells lining up to form brunching lumen and tubes mimicking vascular network allowing extensive nutrition to the tumor mass^58^. Here we clearly show that catulin plays a pivotal role in breast cancer tumorigenesis and invasiveness process. It is a great marker of heterogeneous population of invasive breast cancer cells and by interacting with different cancer stem cell and adherens junction molecules it may be a significant linker between different pathways leading to enhanced cancer cell motility. That is why deciphering the complex nature of this protein is very important. Moreover, here we show that invasiveness of cancer cells is a very complex process linking cancer stem cell theory with vascular mimicry and EMT. While a number of currently used cancer therapeutics are effective inhibitors of angiogenesis, developing a new class of vascular mimicry specific inhibitors could allow for the treatment of angiogenesis-resistant tumors, inhibit cancer metastasis and improve patient survival. Therefore, further investigating in cancer research needs broader perspective combining those processes together.

## Materials and methods

### Generation of stable cell lines and cell culture

MDA-MB-231 and MCF7 cell lines were cultured in DMEM High Glucose (Biowest #L0102-500) supplemented with 10% FBS (Biowest, #S181S-500), 100 IU penicillin and 100 μg/mL streptomycin (Biowest #L0018-100). HCC1806 cells were cultured in RPMI-1640 (Biowest #L0500-500) supplemented with 10% FBS (Biowest, #S181S-500), 1 mM sodium pyruvate (Gibco), 100 IU penicillin and 100 μg/mL streptomycin (Biowest #L0018-100). MDA-MB-468 were cultured in L-15 supplemented with 10% FBS (Biowest, #S181S-500), 100 IU penicillin and 100 μg/mL streptomycin (Biowest #L0018-100). HCC1806, MDA-MB-231 and MDA-MB-468 cell lines were obtained from the American Type Culture Collection (ATCC) and authenticated by ATCC with tests such as short tandem repeat profiling (STR profiling). All cells were maintained in a humidified atmosphere at 37 °C with 5% CO2. pGIPZ lentiviral shRNA clones (two independent shRNA sequences against human catulin) are available from Open Biosystems and were packaged according to manufacturer’s protocol. See Supplementary Materials and Methods for clone details. At 48 h, post-transduction, cells were selected with puromycin to establish stable cell lines. The number of living cells was calculated by Trypan Blue staining using EVETM Automatic Cell Counter (Nano EnTek, South Korea). Cell line was regularly tested for mycoplasma contamination using a PCR-based method (Young et al., 2010).

### Plasmid stable transfection

To generate stable alpha-catulin promoter GFP reporter cell lines MDA-MB-231 and HCC1806 cells were transfected with GLuc-ON Promoter Reporter Clone (GeneCopoeia # HPRM14050-PF02) construct using Lipofectamine 3000 Transfection Reagent (Thermo Fisher Scientific #L3000001) according to manufacturer’s instructions. See Supplementary Materials and Methods for clone details. At 48 h’ post-transfection, cells were selected with puromycin to establish stable cell lines. Fluorescence was examined under confocal microscope LSM 700 (Zeiss).

### RNA isolation, cDNA synthesis and Real-Time Quantitative PCR (RT-qPCR)

RNeasy Mini Kit (Qiagen, #74,106) was used to isolate total RNA from MDA-MB-231 catulin KD and control cells and HCC1806 KD and control cells, according to manufacturer’s instructions. With the usage of DeNovix DS-11 Spectrophotometer concentration and purity of RNA was established. cDNA was synthetized according to the PrimeScript RT Master Mix (TAKARA BIO INC. #RR036A) protocol (1 μg of RNA was used). Catulin gene expression levels were quantified by RT-qPCR. Each reaction mixture was composed of as follows: 1 μl of 20-times diluted cDNA, 6.25 μl of Fast SG qPCR Master Mix (2x) (EurX, #E0411) and 0.5 μM oligonucleotide primers (listed in section Supplementary Material and Methods) in a total volume of 12.5 μl. Reactions were performed in triplicates on the LightCycler 480 II (Roche) in the following conditions: 40 s at 95 °C, followed by 42 amplification cycles (95 °C for 15 s, 60 °C for 15 s, 72 °C for 15 s). Catulin expression level was normalized to GAPDH. Primers are provided in Supplementary Materials and Methods. Statistical significance was determined using student *t*-test. *p* < 0.05 was considered statistically significant. FACS sorted cells were immediately collected in RNAprotectR cell reagent (QIAGEN). Then they were centrifuged and resuspended in RLT buffer according to manufacturer’s protocol. RNeasyR Micro Kit (QIAGEN). Was used to extract total RNA from those cells. RNAseq analysis was performed by the Next-Generation Sequencing core facility in the Centre of New Technologies, University of Warsaw with the usage of NovaSeq 6000 system (Illumina).

### Expression data analysis

The expression and data analysis were performed as described in^56^. The RNA-seq libraries were paired-end sequenced using NovaSeq 6000 at the CeNT’s Genomics Core Facility. The resulting reads were trimmed; Illumina adapters were removed using trimmomatic v0.36. Quality of individual fastq files was assessed by FastQC. Remaining rRNAs were removed using sortmeRNA v3.03 and then mapped to hg38 reference transcriptome (GRCh38.p13 gencode v34) using STAR aligner. Due to the contamination of the samples with mouse transcripts and after careful principal component analysis, 1 control sample was removed from the downstream analysis. Such aligned reads were then quantified using Salmon v0.13.1 Differentially expressed genes (DEGs) were then identified using DESeq2. Only genes of adjusted p-value less than 0.05 were considered.

### Statistical analysis

Statistical significance was determined using student *t*-test. *p* < 0.05 was considered statistically significant.

### Western blot

Cells from the 10 cm plate were scraped with the usage of ice cold PBS, centrifuged and resuspended in 200 μL of lysis buffer containing 0.15 M NaCl, 1% Triton X100, 0.05 M Tris, and protease inhibitor cocktail III (Calbiochem). Cells were multiply passed through a 26 g needle, incubated on ice for 30 min, and then centrifuged at 14,000 × g for 15 min at 4 °C. Supernatant containing proteins was then collected and separated on 4–12% NuPAGE Novex Bis–Tris gels (Invitrogen) and transferred onto a nitrocellulose membrane with a semi-dry Biorad transfer system for 40 min at 70 mA. Membranes were then blocked in a 5% skim milk diluted in TBS-T (TBS with 0.1% Tween-20) for 1 h at RT on a gentle shaker. Primary antibody in 5% skim milk in 0.1% TBS-T was then added to the membranes and incubated on a gentle shaker overnight at 4 °C. Membranes were washed 3 times with TBS-T and secondary antibodies (peroxidase conjugated) were added in 0.1% TBS-T. Membranes were then incubated for 1 h at room temperature on a gentle shaker. Antibody list is included in Supplementary Materials and Methods. Amersham Imager 600 RGB was used to visualize the blots.

### Sphere formation and invasion assay

Cells transduced with non-silencing and catulin specific shRNA and cells transfected with catulin promoter reporter system were used for sphere invasion assay. 1000 serum free cells were seeded in 200 μl of sphere formation medium [DMEM/F12 (Biowest), 1% B27 supplement (Gibco), 20 ng/mL EGF (Invitrogen) and 20 ng/mL FGF(Invitrogen)] in 96 well Corning Costar ultra-low attachment plate and allowed to form spheres for 72 h. Then spheres were mixed with 1:3 matrigel (Corning)—collagen (Corning) solution and plated on Millicell EZ Slide 8-well glass (Merck Millipore). Incubation and invasion was allowed for 48 h. Immunostainings were performed according to Elia., Lippincott-Schwartz., 2009. Sphere invasion assays were repeated independently 3 times. Images were taken using confocal microscope LSM 700 (Zeiss). A one-way ANOVA test was applied to calculate the statistical significance for distance of invasion considering data from all 3 experiments where *P* < 0.05).

### Chip invasion assay

Cells transduced with non-silencing and catulin specific shRNA were used for chamber invasion assay. 3D Cell Culture Chips (#Dax-1, Aim Biotech) were used for invasion assays. 10^6^ of cells were counted and resuspended in serum free medium and were added into media channel of invasion chip. Medium containing chemoattractant (20% FBS) was added to the opposite media channel. In between those two channels there was a hydrogel channel containing 1:3 matrigel (Corning)—collagen (Corning) solution. Chips were maintained in a humidified atmosphere at 37 °C with 5% CO2. Invasion was allowed for 0, 24, 48, 72, 96 h. Invasion assays were repeated independently 3 times. Images were taken using fluorescence microscope Axio Observer (Zeiss). A one-way ANOVA test was applied to calculate the statistical significance for distance of invasion considering data from all 3 experiments where *P* < 0.05).

### Xenograft transplants

5 × 10^5^ of MDA-MB-231 cells transduced with non-silencing and catulin specific shRNA and cells transfected with catulin promoter reporter system, suspended in medium mixed 1:1 with matrigel (Corning) were injected into 4th mammary gland of NOD.CB17/Prkdcscid/scid/Rj mice, using isoflurane as a general anesthetic. Each variant of cells was injected in 5 repeats. Tumors were allowed to form for 4–9 weeks before sacrificing and collecting the primary tumor from each subject. According to the protocol, mice were euthanized with CO_2_ following a cervical dislocation. Briefly, tumors used for RNA isolation were first dissected from the mouse, minced into small pieces, incubated in collagenase (1000U/mL) for 1 h at 37 °C, washed in PBS, trypsinized (0.25% trypsin- EDTA from Gibco) for 20 min at 37 °C, and FACS sorted for GFP + and GFP- cells using a BD Biosciences FACSAria cell sorter. All experiments and procedures were preapproved by the First Warsaw Local Ethics Committee for Animal Experimentation nr 214/2017, 26 April 2017. The animals were treated in accordance with the EU Directive 2010/63/EU for animal experiments, and the ARRIVE guidelines (https://arriveguidelines.org).

### Indirect immunofluorescence detection

Tumors formed in our xenografts were resected washed with PBS and embedded in OCT. Then they were sectioned at 12 μM with the usage of Leica Cryostat for indirect immunofluorescence detection of various markers. At first samples were fixed in 4% paraformaldehyde for 10 min, washed with PBS and permeabilized in PBS-T (PBS with 0.1% Triton X-100) for 10 min. Then the tissues were blocked in 0.1% BSA, 2.5% NGS (normal goat serum), 2.5% NDS (normal donkey serum) in PBS-T for 1 h at RT. Primary antibodies were diluted in 0.1% BSA in PBS-T and incubated overnight at 4 °C. Descriptions and dilutions of primary antibodies are described in Supplementary Materials and Methods. Secondary antibodies conjugated with Alexa Fluor 488 or 594 were diluted 1:500 in blocking solution and incubated for 1 h at RT. Confocal microscope LSM 700 (Zeiss) was used to take pictures.

### Immunohistochemistry

The immunohistochemical staining analysis for Catulin was performed on tissue microarray slide panel BR8014 (Biomax, Derwood, MD, USA). The slide consists of breast cancer and normal tissue microarray, containing 30 cases of carcinoma (26 invasive ductal carcinomas, 4 invasive lobular carcinoma), 5 each of adjacent normal tissue and normal tissue, duplicate cores per case. The procedure was performed as described previously in Karpinska et al., 2021^56^. Briefly, the slide was deparaffinized and rehydrated and after blocking the endogenous peroxidase activity in 0.3% H2O2 for 3 min, the slides were blocked in a blocking buffer (0.1% gelatin, 0.1% BSA, 2.5% donkey serum, 2.5% goat serum, and 0.3% Triton X in PBS) for 1 h at room temperature. Slides were then incubated with a primary antibody in 0.1% BSA in 0.1% PBS-T ON at 4 °C. Next day, the slides were incubated in appropriate biotin-conjugated secondary antibody (1:100) (Vector Labs, Burlingame, CA, USA) for 1 h in the blocking buffer, followed by the incubation in the prepared Vectastain A + B solution (2 drops of A, 2 drops of B, 2.5 mL PBS 1X) for 30 min at RT. Reactions were developed using the diaminobenzi-dine (DAB) reagent as the chromogenic substrate (SK-4100; Vector). The sections were counterstained with 5 × diluted haematoxylin, mounted in 80% glycerol and examined under a light microscope.

### Spheres serial replating assay

Cells transduced with non-silencing and catulin specific shRNA were used for sphere serial replating assay. 20,000 serum free cells were seeded in 1 ml of sphere formation medium [DMEM/F12 (Biowest), 1% B27 supplement (Gibco), 20 ng/mL EGF(Invitrogen) and 20 ng/mL FGF(Invitrogen)] in 6 well Corning Costar ultra-low attachment plates. Cells were allowed to form spheres for 7 days. After 7 days, spheres were trypsinized and counted and calculated by Trypan Blue staining using EVETM Automatic Cell Counter (Nano EnTek, South Korea). 20,000 of cells were then plated again in 1 ml of sphere formation medium and allowed for the next 7 days to form spheres again. Whole process was repeated 3 times until day 28th was reached. The results were calculated as sphere forming capacity = number of spheres/number of cells plated [%]. A one-way ANOVA test was applied to calculate the significance of the number of spheres considering data from all 3 experiments where *P* < 0.05).

### Ethics approval and consent to participate

All experiments were preapproved by the LKE (local ethic committee) at the University of Warsaw.

## Supplementary Information

Supplementary Information.

## Data Availability

The data regarding manuscript are presented in main figures of the article. The datasets generated during the current study are not publicly available due to continuation of the project and preparation of the next publication. Additional data obtained in this study are available on request from the corresponding author.

## Abbreviations

BC: Breast cancer
EMT: Epithelial–mesenchymal transition
VM: Vascular mimicry
GFP: Green fluorescent protein
MCAM: Melanoma cell adhesion molecule
ER: Estrogen receptor
PR: Progesterone receptor
Her2: Human epidermal growth factor receptor 2
hHNSCC: Human head and neck squamous cell carcinoma
GEF: Guanine exchange factor
GTP: Guanosine-5'-triphosphate
CSC: Cancer Stem Cells
CatGFP: Catulin GFP reporter plasmid
KD: Knock down
Ctrl: Control
