# Supplementary material for: Catulin reporter marks a heterogeneous population of invasive breast cancer cells with some demonstrating plasticity and participating in vascular mimicry
Source: Sci Rep. 2022 Jul 25;12:12673. doi: 10.1038/s41598-022-16802-2 (PMC9314412; doi:10.1038/s41598-022-16802-2)
Supplement: Supplementary file 1 — Supplementary Information. [file 41598_2022_16802_MOESM1_ESM.pdf]

Fig. Suppl. 1

a

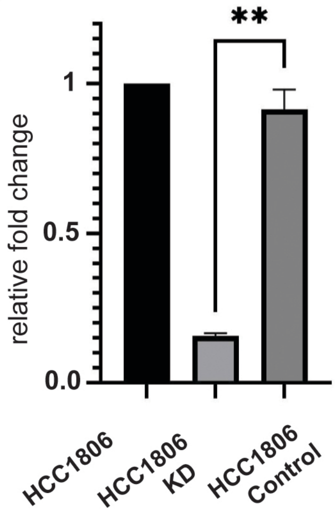

b

position	stage	tnm	IHC staining intensity (average from 2 sections)
A1	IIIb	T4N1M0	1
A7	IIb	T2N1M0	2
A3	IIa	T1N1M0	0
B1	IIa	T3N1M0	1
B5	IIIb	T4N1M0	3
B9	IIb	T2N1M0	3
C7	IIb	T2N1M0	3
C9	IIb	T2N1M0	3
D9	IIb	T2N1M0	2
E5	IIIa	T2N2M0	3
F9	IIb	T2N1M0	2
B3	IIa	T2N0M0	1
A9	IIa	T2N0M0	1
C1	I	T1N0M0	0
A5	IIa	T2N0M0	1
B7	IIa	T2N0M0	0
C3	IIa	T2N0M0	0
C5	IIa	T2N0M0	2
D1	IIa	T2N0M0	2
D3	IIa	T2N0M0	1
D5	IIa	T2N0M0	1
D7	IIa	T2N0M0	0
E1	IIa	T2N0M0	0
E3	IIa	T2N0M0	0
E7	IIa	T2N0M0	3
E9	IIa	T2N0M0	0
F1	IIa	T2N0M0	0
F3	IIa	T2N0M0	0

c

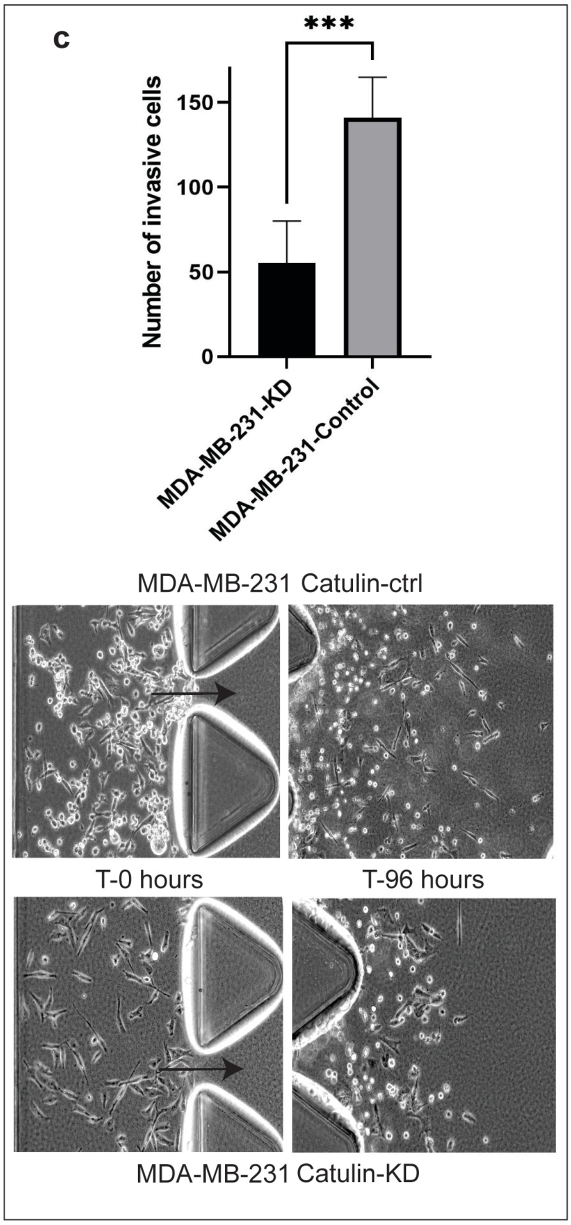

d

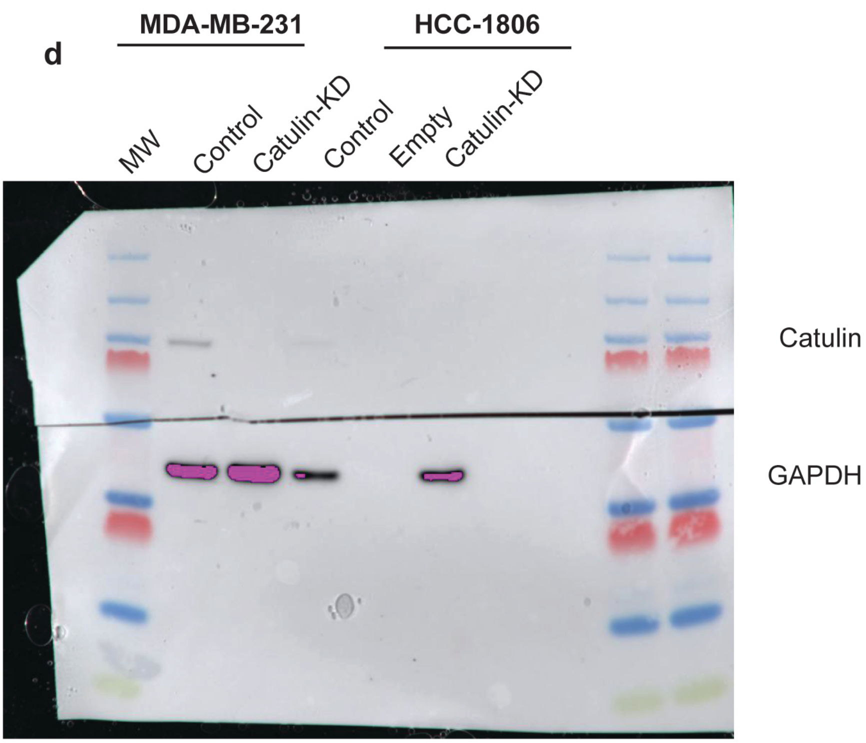

Fig. Suppl. 2

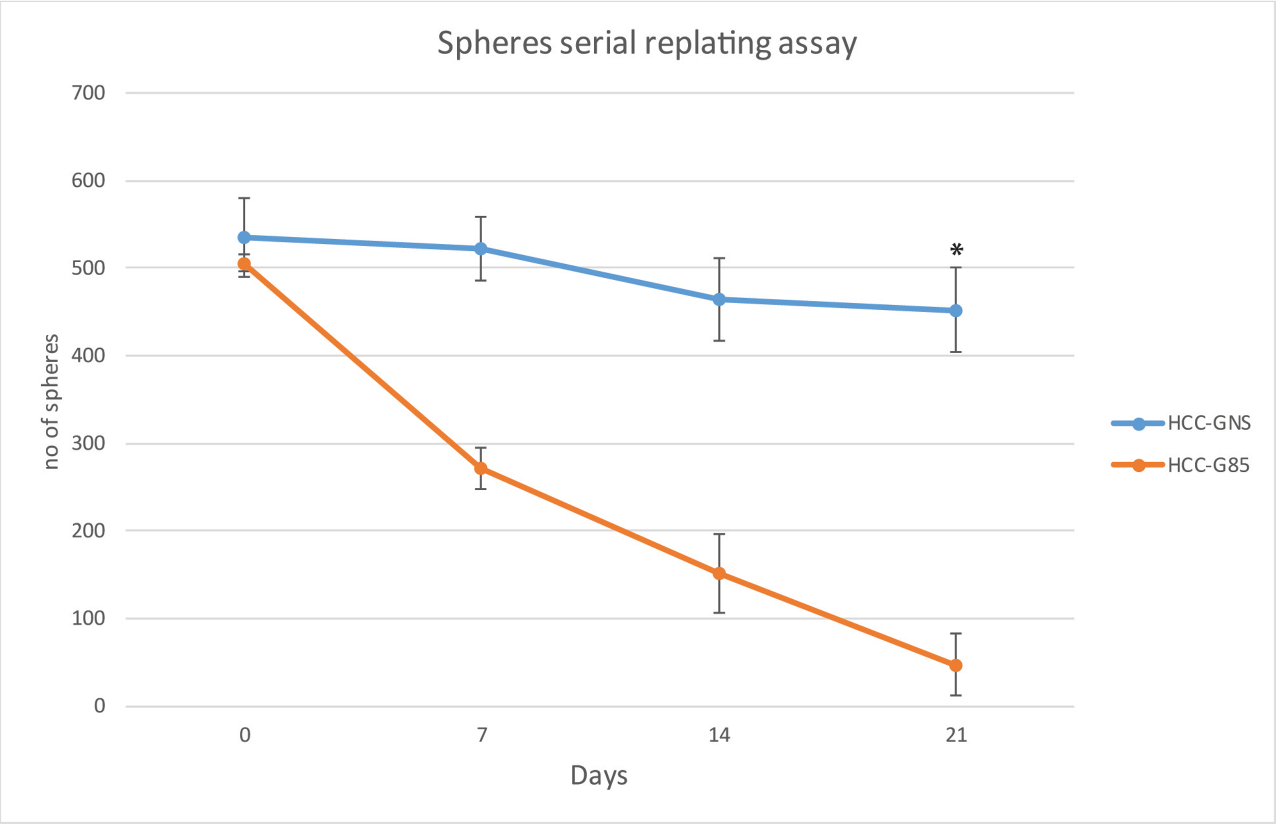

Spheres serial replating assay
700
600
500
400
300
200
100
0
no of spheres
0
7
14
21
Days
*
HCC-GNS
HCC-G85

Fig. Suppl. 3

a **MDA-MB-231 Cat-GFP reporter GFP positive vs GFP negative**

MDA231-CatGFPp-vs-GFPn
PCA-ggplot

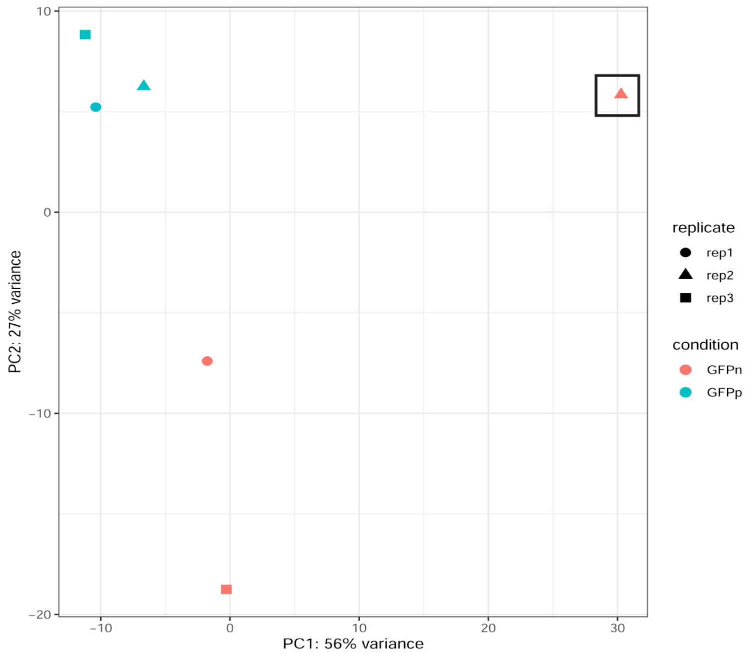

MDA231-CatGFPp-vs-GFPn
noGFPnRep2-Volcano-annotations

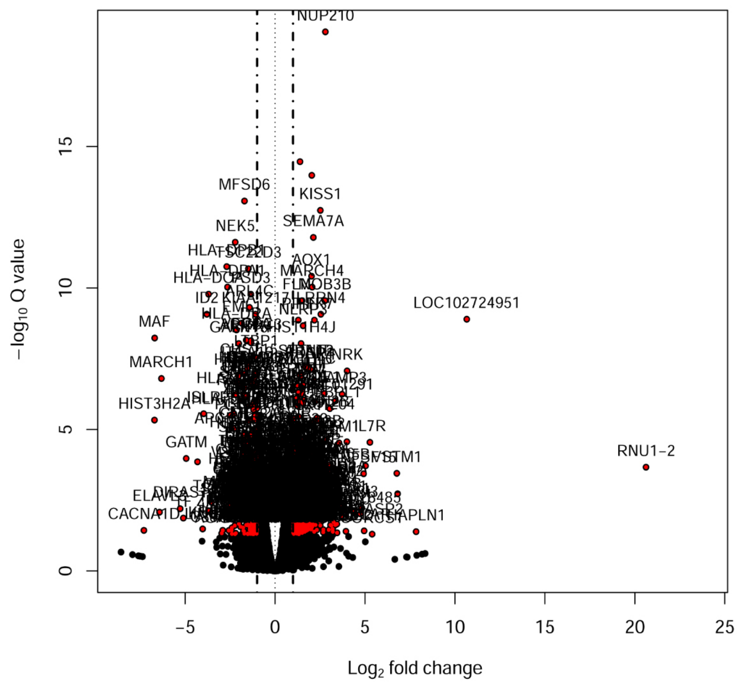

b **MDA-MB-231 Cat-KD (G85) vs CatCtrl (GNS)**

MDA231-in.vivo-G85-vs-GNS
PCA-ggplot

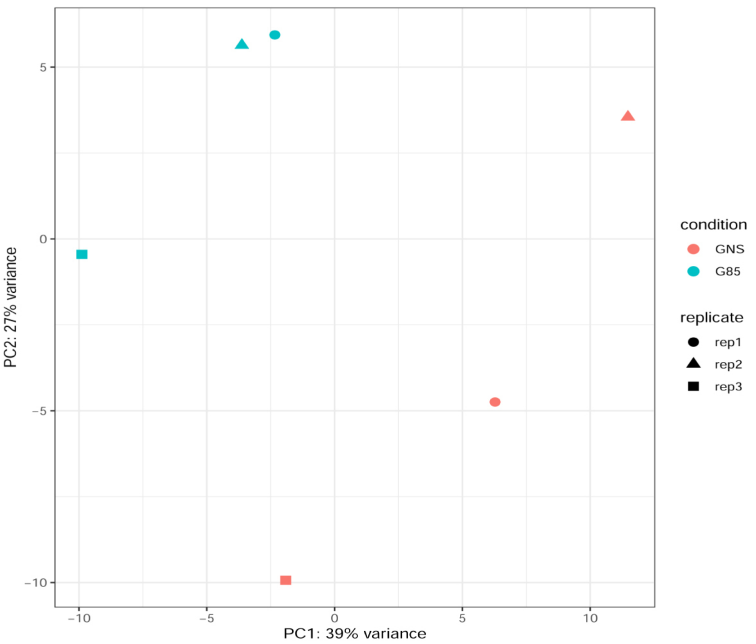

MDA231-in.vivo-G85-vs-GNS
Volcano-annotations

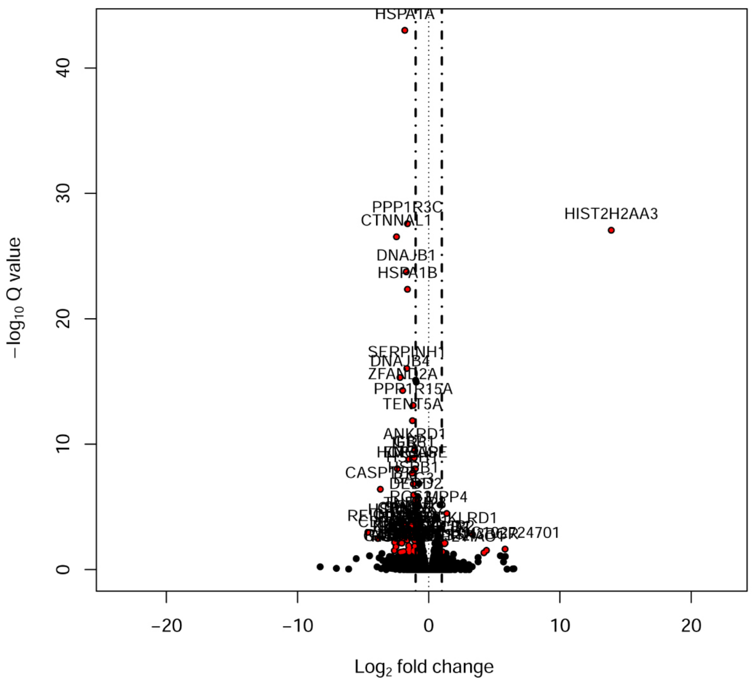

Fig.1S Catulin is expressed in triple negative breast cancer cell lines and in high grade human breast cancer tissue and its ablation in hBC cell lines decreases their invasive potential in the 3D spheroid assay.

a, RT-qPCR shows significant stable knockdown of catulin expression in HCC1806 cells (t-test, p<0,005). b, Table represents immunohistochemistry staining of human breast cancer samples. Tumor samples were divided into two groups: one, advanced group in stages between T2-T4 with lymph nodes involved – N1, second group less advanced in stages between T1-T2 without lymph nodes involved – N0. Average IHC staining intensity included (1-3). c, Invasion chamber assay shows impaired invasive properties of MDA-MB-231 knockdown cells and the chart shows calculated cells that passed through the barrier (t-test, p<0,005). d, Picture of whole WB membrane confirming protein level of catulin and GAPDH in control and knock-downed cells. The membrane was cut in the middle in order to stain with two independent antibodies (against catulin and GAPDH).

Fig.2S Catulin influence breast cancer stem cell potential. HCC1806 cells. Sphere serial replating assay. Sphere forming capacity presented as number of spheres counted on each time point.

Fig.3S RNAseq analysis of catulin dependent genes in highly invasive breast cancer cells. a, Analysis of genes deregulated comparing GFP positive versus negative cells. On the left side, a chart showing PC1/PC2 variance correlation representing 3 independent replications in 2 conditions. 1 negative group marked in black bracket was excluded from further analysis. On the right side, volcano plot showing Log2 Fold Change of all deregulated genes. b, Analysis of genes deregulated comparing Catulin depleted cells (G85) to control cells (GNS). On the left side, a chart showing PC1/PC2 variance correlation representing 3 independent replications in 2 conditions. On the right side, volcano plot showing Log2 Fold Change of all deregulated genes with alpha catulin (internal control) highlighted to be downregulated.

Supplementary Materials and Methods

sqRT-PCR Primers
Catulin-RT-F: 5’ TCGCCTGCATCGAAGCCAAGCAA
Catulin-RT-R: 5’ ACAGCTGGGATGCCTGAGACATGT
GAPDH-F: 5’ GCAAAGTGGAGATTGTTGCC
GAPDH-R: 5’ CCTGCTTCACCACCTTCTTG

Antibodies for Indirect Immunofluorescence and Immunohistochemistry

**Antibody**	**Dilution**	**Company**
β-Catenin	1:300	Sigma-Aldrich #C2206
CTNNAL1	1:200	Abcam #ab96184
CD31 (PECAM)	1:100	Abcam #ab9498
CD44	1:50	BD Pharmingen #550392
CD24	1:50	BS Pharmingen #555428
CD146 (MCAM)	1:200	Abcam #ab75769
GFP	1:4000	Abcam #ab13970
Vimentin	1:400	Abcam #ab92547

Antibodies for Western Blot

**Primary Antibody**	**Dilution**	**Company**
CTNNAL1	1:1000	Abcam #ab96184
GAPDH	1:1000	Abcam #ab9485

**Secondary Peroxidase Conjugated Antibody**	**Dilution**	**Company**
Anti-mouse	1:1000	Sigma-Aldrich #A9044
Anti-rabbit	1:1000	Sigma-Aldrich #A0545

Expression data analysis

The RNA-seq libraries were paired-end sequenced on NovaSeq 6000. The resulting reads were processed as in (Zhang et al., 2017). Briefly, the reads were trimmed, Illumina adapters were removed using trimmomatic v0.36 (Bolger et al., 2014) using the following options: LEADING:20 TRAILING:20 SLIDINGWINDOW:6:20 MINLEN:75 CROP:100. Quality of

individual fastq files was assessed by FastQC (Andrews et al., 2010). Subsequently, remaining rRNAs were removed using sortmeRNA v3.03 (Kopylova et al., 2012) and then mapped to hg38 reference transcriptome (GRCh38.p13 gencode v34 (Frankish et al., 2018)) using STAR aligner (Dobin et al., 2013). Due to the contamination of the samples with mouse transcripts and after careful principal component analysis, 1 control sample was removed from the downstream analysis. Such aligned reads were then quantified using Salmon v0.13.1 (Patro et al., 2017) using the following options: --validateMappings --rangeFactorizationBins 4 --seqBias --gcBias --numBootstraps 100. Differentially expressed genes (DEGs) were then identified using DESeq2 (Love et al., 2014).

Andrews, S., Krueger, F., Segonds-Pichon, A., Biggins, L., Krueger, C., and Wingett, S. (2010). FastQC.

Bolger, A.M., Lohse, M., and Usadel, B. (2014). Trimmomatic: a flexible trimmer for Illumina sequence data. Bioinformatics 30, 2114–2120.

Dobin, A., Davis, C.A., Schlesinger, F., Drenkow, J., Zaleski, C., Jha, S., Batut, P., Chaisson, M., and Gingeras, T.R. (2013). STAR: ultrafast universal RNA-seq aligner. Bioinformatics 29, 15–21.

Frankish, A., Diekhans, M., Ferreira, A.-M., Johnson, R., Jungreis, I., Loveland, J., Mudge, J.M., Sisu, C., Wright, J., Armstrong, J., et al. (2018). GENCODE reference annotation for the human and mouse genomes. Nucleic Acids Res 47, gky955-.

Kopylova, E., Noé, L., and Touzet, H. (2012). SortMeRNA: fast and accurate filtering of ribosomal RNAs in metatranscriptomic data. Bioinformatics 28, 3211–3217.

Love, M.I., Huber, W., and Anders, S. (2014). Moderated estimation of fold change and dispersion for RNA-seq data with DESeq2. Genome Biol 15, 550.

Patro, R., Duggal, G., Love, M.I., Irizarry, R.A., and Kingsford, C. (2017). Salmon provides fast and bias-aware quantification of transcript expression. Nat Methods 14, 417–419.

Zhang, C., Zhang, B., Lin, L.-L., and Zhao, S. (2017). Evaluation and comparison of computational tools for RNA-seq isoform quantification. Bmc Genomics 18, 583.
